# Using 'may contain' labelling to inform food choice: a qualitative study of nut allergic consumers

**DOI:** 10.1186/1471-2458-11-734

**Published:** 2011-09-26

**Authors:** Julie Barnett, Kate Muncer, Jo Leftwich, Richard Shepherd, Monique M Raats, M Hazel Gowland, Kate Grimshaw, Jane S Lucas

**Affiliations:** 1 Department of Information Systems and Computing, Brunel University, Uxbridge, Middlesex, UB8 3PH, UK; 2 Department of Psychology, University of Surrey, Guildford, Surrey, GU2 7XH, UK; 3 Allergy Action/Anaphylaxis Campaign, Farnborough, GU14 6SX, UK; 4 School of Medicine, Division of Infection, Inflammation and Immunity, University of Southampton, Tremora Road, Southampton, S016 6YD, UK

## Abstract

**Background:**

Precautionary 'may contain' warnings are used to indicate possible allergen contamination. Neither food safety nor foods labelling legislation address this issue. The aim of this study is to understand how peanut and nut allergic adults interpret 'may contain' labelling and how they use this information when purchasing food.

**Methods:**

Qualitative methods were used to explore both behaviour and attitudes. The behaviour and 'thinking aloud' of 32 participants were recorded during their normal food shop. A semi-structured interview also explored participants' views about 13 potentially problematic packaged foods. Transcribed data from these tasks were analysed to explore the interpretation of 'may contain' labelling and how this influenced food choice decisions.

**Results:**

Peanut and nut allergic individuals adopt a complex range of responses and strategies to interpret 'may contain' labelling. Many claimed such labelling was not credible or desirable; many ignored it whilst some found it helpful and avoided products with all such labelling. Interpretation and consequent decisions were not only based on the detail of the labelling but also on external factors such as the nature of the product, the perceived trustworthiness of the producer and on the previous experience of the nut allergic individual.

**Conclusions:**

'May contain' labelling was interpreted in the light of judgements about the product, producer and previous personal experience. It is vital that these interpretation strategies are taken into account by those responsible for labelling itself and for the provision of advice to nut allergic individuals. Suggestions to improve labelling and advice to the allergic individual are considered.

## Background

Approximately 1% of North American and UK populations are allergic to peanuts and other nuts [1-3] and these foods cause the most frequent severe and fatal food associated reactions [4-6]. At present, peanut or tree nut allergy (from now, jointly referred to as 'nut allergy') cannot be treated and management involves avoidance of nuts and emergency treatment of reactions; the aim being to reduce morbidity, mortality and improve an allergic individual's quality of life [7-9].

Consumers face complex food choices on a daily basis. Most consumers balance a number of issues when considering which foods to eat, including the price, taste and whether the food is nutritious [10,11]. Food allergic individuals additionally need to avoid allergens to prevent potentially life threatening reactions. Packet information is vital in assisting with such decisions.

To help nut allergic consumers avoid products that contain nuts, there are several sources of information on food packaging. These include the product name, ingredients list, allergy ('contains') advice and precautionary ('may contain') information. If there is a possibility that a food may contain traces of an allergenic food, not as an intentional ingredient, but as a result of cross-contamination through, for example, shared manufacturing equipment, this risk is often indicated by a precautionary 'may contain' type label (e.g. 'may contain traces of peanut'). Such precautionary statements are not covered in either food safety or food labelling legislation [12]. In a U.S. supermarket survey of over 20,000 products [13], 17% of products had precautionary allergen labelling. In a study from 10 European countries of over 500 types of biscuits and chocolate, 'may contain' type labelling was included on the packaging of 26% of biscuits and 80% of chocolate. In addition 31% of the biscuit labels referred to the possible presence of allergens in the production environment and 6% of the chocolate labels did so [14].

There is considerable variety in the actual wording used in may contain labelling (MCL) [15]. The aforementioned supermarket survey [13] noted 25 different variants of MCL in use. The amount of nuts may be more or less specified (e.g. 'nuts' or 'traces of nuts') and the type of nuts may be more or less specified (e.g. 'nuts', 'peanuts' or 'hazelnuts' etc.). 'May contain' messages may also be implied through alternative wording such as 'cannot guarantee nut free'. It may also refer to characteristics of the food production situation e.g. 'produced on a line which also handles nuts'.

The prevalence and variation of precautionary labelling, although intended to assist the consumer in their food choices, is increasingly considered as problematic for food allergic consumers. Expert advice in this area [16] is that consumers should always take seriously any 'may contain' warnings on the label unless they have been advised otherwise by their allergy consultant. The literature suggests that making decisions on the basis of MCLs is problematic in several ways.

Firstly, foods that carry precautionary labels may actually be safe to eat and therefore consumers may be following unnecessarily restrictive diets by heeding the warning labels [17]. It is likely that 90% of products with cautionary labels will contain no residues of peanut protein, and some of those that do are at levels unlikely to cause a clinical reaction [17].

Secondly, consumers are aware of the proliferation of these warnings and, in the absence of a previous allergic reaction to foods labelled in this way they may exercise less caution with foods containing precautionary labelling, with a consequent risk of reaction [18]. A recent study of allergic individuals and their parents reported that 8% of those with accidental reactions attributed it to having ignored a precautionary label [19]. Thirdly, the variation in wording used by different manufacturers for these precautionary labels, although not intended to convey different degrees of risk, is often interpreted by the consumer as doing so [20,21]. A survey of parents of nut allergic patients reported that products labelled 'not suitable for nut allergy sufferers' or 'may contain nuts' were more likely to be avoided than those stating 'may contain traces of nuts' or 'cannot guarantee this is nut free' [22].

Previous research that has explored consumer reactions to MCL and identified these issues is almost entirely quantitative. This has been helpful both in characterising consumer responses to MCL, and in indicating the prevalence of these responses. However, qualitative work is able to provide unique insights into the complex reasoning processes that underlie such responses [23]. Accordingly the present study builds on qualitative work that explores MCL with adolescents and the parents of allergic children [24,25] by focusing on the responses of *adults *with nut allergies. Importantly, the qualitative methods used in this study allow a focus on responses to MCLs in actual shopping situations and in relation to particular products. The chosen combination of methods thus provided the opportunity to explore the research questions in full recognition of the situational and contextual nature of food choice behaviours.

The aim of this study was to explore the food choice decisions made when nut allergic adults are faced with 'may contain' labelling and the way in which they make sense of such labelling.

## Methods

### Study population

Thirty two participants aged 16 or over with a clinical history compatible with IgE-mediated reactions to peanuts and/or tree nuts were recruited to the study. Participants were recruited via letter or email from three sources in the UK: (1) specialist allergy clinics at Southampton University Hospital Trust (SUHT), (2) from one of three primary care settings or (3) from staff and students of the University of Surrey who had received medical care from a mixture of primary, secondary and tertiary care. Potential participants completed a postal screening questionnaire. With the exception of oral allergy syndrome to fruits and/or vegetables (as this would not affect the purchase of labelled products), individuals with allergies or intolerance to foods other than peanut or tree nuts were not admitted to the study. A further 22 participants were eligible to take part in the study but did not wish to (a response rate of 59.3%). Using a classification previously used for peanut allergy [26], participants were classified by an allergen consultant (JSL) in relation to the severity of their worst ever reaction to nuts (severe, moderate or mild). The study was approved by the National Research Ethics Service and University of Surrey Ethics Committee.

Participants took part in three tasks: an accompanied shop, followed by an interview which also included a product choice reasoning task. This triangulation of methods was undertaken in order to "reveal the different dimensions of a phenomenon and to enrich understandings of the multi-faceted, complex nature of the social world" [27].

### Accompanied shop

Participants were firstly trained in a 'think aloud' methodology [28,29] and then observed during their normal food shopping trip [30-32]. Whilst shopping, participants were asked to talk aloud at all times about what they were thinking with regard to their food choices. The researcher used prompts such as "what are you thinking now?" and did not enter into conversation with the participant during the shop. Important behaviours and comments made by the participant were followed up in the interview.

### Semi-structured interview

Following the accompanied shop, an in-depth semi-structured interview was carried out in the participant's home. The first part of the interview sought participant reflections on the accompanied shop. The interview schedule explored various aspects of living with nut allergy such as diagnosis, symptoms, management of nut allergy before focusing on their views about product labelling in general and MCL in particular.

### Product Choice Reasoning Task (PCRT)

As part of the interview, before being asked about labelling, participants took part in the PCRT. They were shown 13 food products believed to pose potential dilemmas for nut allergic consumers. Some of these dilemmas related to 'may contain' type labelling. Products were assigned by an allergy dietician as belonging to product categories that would generally be designated as high or low risk to nut allergic consumers independently of what information was presented on the label. There were 'may contain' type warnings on 6 of the PCRT products. Participants were asked if they would eat each food item and were asked to identify the factors that led them to make these decisions and the sorts of dilemmas they may have experienced in doing this. Details of the products used in the PCRT can be found in Barnett et al. [12] and a full description of all the methods and their protocols can be found in Barnett *et al*. [33].

### Analysis

All parts of the study were recorded and fully transcribed. This provided a rich and detailed corpus of data for analysis. The transcripts were coded by two researchers using NVivo qualitative data analysis software [34]. Established techniques of thematic coding [35] were used to capture the key points, positions and opinions that were expressed. Areas of both consensus and difference were identified in order to assist with characterising and interpreting each theme. The developing interpretations were regularly revisited by the researchers responsible for the analysis on a day to day basis (JB, JL and KM) and these were checked with the broader research team in order to confirm the validity of the interpretations that were being developed. This team included a clinician (JSL) and the project advisor who has a severe nut allergy (MHG).

Quotes are used below to illustrate the analytic points being made and the participants who provided each quote are identified in relation to which method the quote was produced (AS = accompanied shop, I = interview, PCRT = product choice reasoning task) their gender (F = female, M = male) and severity of allergy (Severe, Moderate or Mild). Where the quote includes an utterance by the Interviewer this is indicated using I for Interviewer and P for Participant.

## Results

### Study participants

Thirty two respondents were eligible and consented to participate within the time-frame of the study (9 male; age range 16-70 years). Twenty two participants were recruited from SUHT specialist allergy clinics, 4 from primary care settings and 6 from University staff and students. Eighteen participants described previous severe reactions, 12 moderate and 2 mild. Five participants had peanut allergy, 9 tree nut allergy and 18 had both peanut and tree nut allergies. On average, participants had had a diagnosed nut allergy for 20 years (range 1- 63 years). Twenty two reported suffering from a reaction within the last 2 years.

### Evaluating and using 'may contain' labelling

Firstly we will outline participant evaluations, both positive and negative, of MCL. Secondly, we consider how participants used MCL to decide whether to purchase particular foods.

#### 1. Evaluations of 'may contain' labelling

MCL was generally considered preferable when compared to receiving no information regarding the possibility of risk. However, there was extensive evidence that participants discounted the 'may contain' message in a range of ways. The view that MCL was neither credible nor desirable was frequently expressed.

MCL was seen to be important when the food product was considered potentially risky. Under these circumstances the uncertainty of having no information about potential cross contamination was viewed as unsatisfactory.

*Even in...even in places like (coffee shop), there's a little note on the sign that says, "This contains nuts," tick. There's not even that in the (supermarket) bakery section. So it would help to have something which says "This may contain nut traces", even though I hate it when it says "may contain". Even that would be better than nothing*. (I, M, Severe)

MCL was also seen as positive when compared with the lack of nut warnings evident on foreign products.

*But they must be a comfort because that's why I feel nervous about the foreign packaging that doesn't have anything. So even though they might, you know, consciously, you might not really think they're much use, but subconsciously, they're giving you some kind of support. They're like a comfort to you. You feel like, oh, people are checking my food *(I, F, Moderate)

MCL may be seen to provide reassurance as it conveys the message that the nut content of food products has been assessed and attended to by the manufacturer.

Most participants however claimed that the 'may contain' message was not a helpful one. There were 4 main ways in which participants discounted the 'may contain' message. First, some considered that it was not possible to avoid all products with MCL and that doing so would result in an unfeasibly limited diet.

*I've now become sort of blasé in the millions of things that say because if I didn't eat things that said "may contain traces of nuts" I'd have a very narrow spectrum of food that I could eat *(I, M, Severe)

They therefore discounted the 'may contain' message for pragmatic reasons.

Secondly, other participants felt that the motivations of the message source (manufacturers or retailers) are suspect and thus that the message is not to be trusted. The main motivation imputed to such sources was that they were simply trying to avoid any liability should any adverse reactions occur.

*I can understand why (the 'may contain' messages) are there, because it's a backside-covering exercise for the manufacturers, because they can say "Well we put it may contain traces of nuts in it, and he died, so it's not our fault". So I can see why they've done it, but it's the over-usage of it - it's the boy who cried wolf syndrome. After a while you just become blasé to it and you just go, "well I'm going to eat it anyway"*. (I, M, Severe)

*If it says "may", I generally trust it and I generally buy it, but ...That's how they cover themselves in the manufacturing process isn't it? *(AS, M, Severe)

Interestingly in the second quote above the participant equates trusting MCL with the notion that there is nothing in the product that he should be concerned about. The impact of trust is complex. In the quote below the participant explains that he is disregarding the MCL as, if there really *were *traces of nuts, the supermarket he shopped in and trusted would warn about this more clearly.

*Right... Now, I'd usually be a bit cautious with this kind of stuff, but, being (supermarket name), I actually trust them quite a lot because they'll probably have a breakdown...[on everything they make]. Oh, it says "May contain traces of nuts" but...I think that actually...they're probably just writing that and actually they... Plus, I have had cookies from (supermarket name) before, so I usually know they're fine. I think actually they would go further - if there was a genuine risk of having nuts in, they would go further than saying 'May contain nut traces.'..*. (AS, M, Moderate)

Thus his trust in the supermarket, along with his experience of the brand, allowed him to discount the veracity of the 'may contain' warning. In the following quote, as the company is trusted, this is taken to mean that where they *do *label with 'may contain' the inference that there is a real risk is warranted.

*I don't know why - I do tend to trust the company if it doesn't put "May contain traces of nut", because so many companies, like (brand name) just chuck that on all their labels, and it makes me then wary of eating it because it says "May contain"*. (I, F, Mild)

Thirdly, participants also attributed different levels of risk to different variants of MCL and considered that different wordings of 'may contain' justified different avoidance strategies: paying attention to the 'stronger' variant of MCL provided grounds for dismissing the 'weaker' version. The first quote below illustrates how 'may contain nuts' was taken more seriously than 'may contain traces of nut' and in the second quote mention of a specific nut was considered to signal a greater risk.

*May contain nuts" is...well, I wouldn't eat it, because that means it could contain nuts. "May contain traces of nuts" is different*. (I, F, Moderate)

*If it says "May contain traces", I'm okay with that - I'll buy that. But if it says quite specifically "May contain traces of peanut", then I won't buy it, because I think that's the... I feel like - I don't feel so confident I think, because I think that's a little bit too specific, you know? *(I, F, Severe)

More specific warnings are read as indicating that there is some particular knowledge about the increased risk of the presence of allergens and participants were more likely to be inclined to take precautionary action accordingly.

Finally there was evidence that nut allergic individuals discounted MCL when they considered them to be implausible. We identified two types of 'implausible discounting': where 'may contain' was located on products that legitimately contained nuts and on products where it was considered impossible that they would contain nuts.

*Well, I mean, when you look at...if you look at a packet of peanuts and it says "This product may contain traces of nuts," it just...the whole thing becomes a joke, doesn't it? That's just silly. You can't put on a packet of nuts "May contain nuts". It's a packet of nuts! You know, if you're going to put that, it just...it seems like another tick-box exercise to reach a standard. It's not actually commonsense*. (I, F, Moderate)

*Well, I just think it's a bit stupid because may contain...well, "ingredients - cannot guarantee..." for like a bottle of lemonade or cherryade or something, is like ludicrous. And they know...they know it's pretty much going to be fine. Yeah, you do kind of ignore them, because you think, if they're just saying that about ingredients on lemonade, maybe that's just going to be the same on ingredients of like a sandwich or something*. (I, F, Moderate)

Interestingly the second quote above suggests that the participant uses the extreme case of flawed MCL to warrant the claim that other products with a much greater likelihood of containing nuts, are not going to be problematic. This extreme form of labelling was considered particularly damaging by people with severe allergies for whom ingesting allergens was particularly dangerous and who consequently endeavoured to take MCL seriously. In such situations adding highly implausible 'may contain' warnings was seen as adding insult to injury to people who could not afford to discount them.

#### 2. Using 'may contain' labelling

There was a broad range of behavioural responses to MCL. A minority of participants said they would always avoid foods labelled with 'may contain'. The majority of participants were at the other extreme and ignored 'may contain' labelling when making a decision to purchase a product. Sometimes participants adopted more differentiated approaches.

##### Avoiding food with 'may contain' labelling

Three participants were clear in their claims that they avoided, and would not eat, products labelled with any variants of 'may contain'. Any indication that nuts could be present led to categorical avoidance of the product.

*To me, if it says "may contain", it means that that person who's produced it isn't sure, and if that person isn't sure and cannot guarantee that it is, then I'm not going to take that chance, simple as that *(I, M, Severe)

MCL was sometimes linked to anxiety. One younger participant described his lack of confidence in dealing with 'may contain' labelling which had resulted in him being too nervous to try new products since moving away from home. He felt that this had constrained his diet to such an extent that he was no longer eating a balanced diet.

*"I hate it when it says "May contain nut traces" because that sentence comes up on pretty much every product, food product. ... I can't walk up to something new and think..... that will be fine, because there's a notice on it which says "May contain nut traces." So that limits what I can try, .. so it's hard to try new things....it's very difficult for me, as an individual, to know what to do basically, you know, how to go about trying new things"*.

*So far, I have to admit, I just haven't tried new things. So far, I've stuck to chocolate muffins, which I can eat, fruit and veg, which is an obvious no nuts, and my parents made me ready-made meals, but I probably go for ready-made meals as well, and just the basics, literally the basics, but I need to obviously expand a bit if I want to...be able to feed myself properly *(I, F, Severe).

Sometimes avoidance was linked to having had a reaction to food labelled with a 'may contain' warning. The fact that they had had a reaction to such products made some participants more wary about consuming may contain products in the future - either all 'may contain' products or the specific product category.

I Have you ever had a reaction?

*P Yes, once, with one that said "May contain traces of nuts"*.

I What was that to?

*P It was a type of chocolate bar from (supermarket) and I had a reaction to it, even though it just said "may contain", and then I never ate anything that said "may contain" again*. (I, F, Severe)

*Yeah, I suppose pasta sauces in a jar. That's why I said I don't eat them, because they've got the warning on, "Produced in a factory that contains nuts" or "May contain nuts", but they don't list nuts in the ingredients, so I've risked it, but had a really mild reaction, so that it's not worth the risk*.

(I, F, Moderate)

##### Ignoring may contain labelling

All three methods used in this research provided evidence that many participants ignored MCL in the sense that they sometimes bought and consumed products labelled in this way. Participants justified their decisions to consume products with MCL in a range of ways and this was not systematically linked to the characteristics of participants such as age, gender or severity of allergy. Some participants thought there was almost no risk involved in consuming products with a 'may contain' warning. For these people MCL was equated with non-existent or imperceptible risk.

*The Carrot & Coriander (soup) is going cheap, so I think that one...that one will be a good one. I've had this before, so I know that will be fine for nuts. No suggestion that there's any nuts in it anyway. Obviously, it says "May contain nut traces", but it won't, so...! *(AS, M, Moderate)

For others the uncertainty MCL signalled was so extensive that taking precautionary action was not warranted and taking a risk was a more preferable approach.

*P "This product is made in a factory which also handles nuts." That statement has absolutely no impact on me, because it doesn't tell me what nuts. In a factory? Is it on the line or just the factory? It's a completely useless statement, as far as I'm concerned*.

I So what do you do when you see that statement usually?

*P I'll just...if it's not in the list of ingredients, I'll just risk it*. (PCRT, F, Severe)

Other participants considered that although there could be a contamination risk they were happy to eat the product anyway.

I So what about (supermarket name) Cauliflower Cheese?

*P They've actually said "no nuts", so at this point, I'd be going I think I'm fine. The recipe has got no nuts. "Ingredients - cannot guarantee nut-free... I'd eat that*.

I Okay, cool. So it's because the recipe thing says no nuts?

*P Yeah, there's a conscious thing there that says they haven't got any nuts in this recipe. There's an off-chance that some nuts might have crept in. There's an off-chance a jumbo jet could land on my head, yeah, but..*. (PCRT, M Severe)

It is noteworthy that such participants accepted that 'may contain' did indicate that the food could indeed contain nuts. The concept of risk, and the importance of running a risk (however small), was prominent here.

The reasoning of other participants involved reference to the potential consequence of having an allergic reaction to nuts. One model that these participants adopted was that they would stop consuming 'may contain' foods as and when they got a reaction.

*The day that I eat something with that warning on that sets me off with a reaction will be a really sad day, because it will mean it will rule out a lot of other stuff that I've [been willing] to risk, but so far, touch wood, most things I eat, if ..., rather than being a bag of oranges or whatever, you know, they all have that caveat, so I just have to disregard it. It's like, yeah, I know that - tell me something else, kind of thing*. (I, F, Severe)

*They'll do. £1.85. These are okay. .... I'm going to keep getting it until I get a reaction, in which case I'll stop! *(AS, F, Severe)

An alternative model was that the likely reaction would be minor and of an acceptable magnitude.

*Yeah. Most of the time, anything that says "May have traces of nuts", then I...if I really want it, then I'll have it, and to be honest with you, you know, that is so low that I'd need to eat a lot of it for it to make me really sick*. (I, F, Severe)

*I mean, I think, really, there needs to be a test for this, because...a simple test that they can say that it's contaminant-free or so minimal... And for me as well, because I have this slight reaction that I know I can get away with it, I just have a really uncomfortable day - I sometimes wonder how far it's going to go, but then drink plenty and it seems to go away. I know I can risk it*. (I, M, Moderate)

##### Interpreting 'may contain' labelling

Participants made sense of MCL with reference to different dimensions of the context in which they are managing their allergy. They judged, interpreted and made use of product packaging information in relation to the two broad dimensions of context described below.

Firstly MCL is interpreted against the backdrop of the participant's experience. Previous experience of a product was an important arbiter of how any uncertainty introduced by MCL was interpreted. Previous experience was trusted to ensure future safety. For one participant the 'may contain' warning could be safely ignored in the light of previous - uneventful - consumption.

*Regarding these, I will always look on the back. It says "No nuts - cannot guarantee nut-free," and I know that's fine because I've had them before*. (AS, M, Severe)

For another participant the experience of the moment, for example being hungry and being in a rush, occasioned a more relaxed approach and 'may contain' products were consumed.

*It all comes down to how hungry you are, what a hurry you're in and everything else. You know, like tonight, if I'd gone to get biscuits and I'd looked at the first lot, and then I think, well, just got to, sod it, I can't, you know, I just haven't got the time, and it does come down to time and sometimes you just have to grab things and run the risk, and other times, you just think I won't bother. I think I'm normally in the category of I won't bother*. (I. F, Severe)

Personal preferences were also important - participants were more willing to eat foods they liked with a 'may contain' label than those they did not like.

*I would say, personally, if I really liked the product, then I would take the risk and eat it*. (I, F, Severe)

In this situation the immediate benefits outweighed potential risk of consuming the food.

Secondly, beliefs about particular products or food groups/product categories can affect the interpretation of MCL. For example, 'may contain' warnings were interpreted as more credible and as warranting avoidance, if they were linked to a 'problematic product'.

I And then there's the issue of the "May contain nuts". So, if something said "May contain traces of nuts", do you find that helpful?

*P It depends on the product. If it's something like (product name) then it wouldn't be an issue. If it's a cereal, like (product name), and it looks a bit dodgy, then I wouldn't entertain it*.

I So does 'may contain' actually help you to make a judgement then or...?

*P Yes, on some products. Like (product), no, I would automatically buy, wouldn't be a problem, but on something I wasn't sure of, like these rice bars or whatever, then I wouldn't buy it*. (PCRT, M, Severe)

This sometimes meant that the look of the product aroused suspicion or if the participant thought it feasible that the product could contain nuts.

In summary then, although there were circumstances when MCL was seen as valuable, participants discounted its veracity in several ways. Not consuming food with MCL was considered unfeasible in the light of their ubiquity, and the perceived motivations of the source rendered the labels untrustworthy. Labels were interpreted to allow ostensibly weaker versions of the 'may contain' warning to be dismissed and the location of warnings on implausible products was also used to justify dismissal. It was clear that for a few, avoiding all foods with MCL was the preferred option and that not to do so caused anxiety. Most participants however, claimed that they *did *eat foods with MCL and were prepared to run the risk at least until they had a reaction. Previous experience was trusted to signal future safety and present affective states and preferences also provided justification for consuming foods with MCL.

## Discussion

This study has explored the food choice decisions made when nut allergic adults are faced with 'may contain' labelling. It is a complex picture. On the one hand many participants did not believe that the 'may contain' message was credible or desirable and ignored it when making food choices. A small number of participants avoided all products labelled in this way. In between there were a range of other evaluations and actions: MCL triggered anxiety and yet could reassure, it was linked both to trust and distrust; it was ignored by some and yet others attributed meaning to minor nuances in the wording. These findings did not seem to be linked to characteristics of the participants such as the severity of their allergy.

This study has provided insights, not only into how people reflect on MCL in the context of an interview but also into ways in which people refer to MCL both in the context of actual shopping practices when making decisions about purchasing food and when faced with particular products. The insights derived from the juxtaposition of 3 different qualitative methods proved of particular value in exploring what allergic individuals actually do, rather than simply working with a more general and decontextualised focus on what nut allergic individuals say. The triangulation of the accompanied shop data where participants were making decisions and balancing multiple purchase considerations in the shopping environment, with data from the subsequent interview and product choice reasoning task enabled a 'thick' [36] and rich description of the purchase and reasoning practices of nut allergic consumers.

Consistent with previous studies [17,20,22] participants valued some versions of MCL more than others. People who are managing allergy on a day to day basis are often and understandably sensitive to the ostensibly small and often meaningless cues that are used in product labelling. However, our findings indicate that the ways in which these cues are interpreted have implications not only for participants' confidence in their risk assessment management strategies but also for the actual product choices that they make. The evidence that the wide variety of formats for providing precautionary information currently leads to nut allergic individuals choosing or rejecting foods on the basis of minor (and meaningless) variations in wording suggests that current guidance recommending standardised wording of 'may contain' type labels is not being followed and might usefully be backed up by legislation.

Hefle *et al *[17] suggested that consumers may be following unnecessarily restricted diets by heeding MCLs. Certainly most participants in this study considered it to be impracticable and undesirable to do so. There were a number of strategies which provided 'rules of thumb' as to when MCL should be heeded or not. Previous experience of consuming a product with no adverse consequences was a major source of reassurance that future consumption would also be uneventful [see too 18]. On the other hand the immediate every day demands of the moment - being hungry or in a hurry for example - gave reasons for greater risk taking.

Adjudged characteristics of either the product or the product provider are also a source of 'evidence' to allergic consumers as to whether or not it is safe to consume products with MCL. In common with other work in this area [24] a common justification for completely ignoring MCL was that it simply represented a strategy for manufacturers to 'cover their backs'. On the other hand MCL may be seen by some nut allergic consumers to provide reassurance - especially when compared with no labelling at all.

The qualitative data collected in this study have enabled an approach that has sought to prioritise the perspective of people with nut allergy and the meanings that they ascribe to the may contain labels that they encounter on a day to day basis in shopping environments. Whilst there is no straight forward application of the concepts of reliability and validity to qualitative research, we have sought to apply appropriate criteria to the conduct and reporting of this research: sensitivity to the setting in which nut allergic people encounter MCL; commitment and rigour in both the data collection and analysis, transparency and coherence in the arguments articulated and the impact and importance of these findings for policy and practice [37]. We recognise that the sample size did not allow us to systematically explore differences between sub-sets of participants in how they made sense of MCL e.g. between different allergy severities, between those with a recent and longstanding diagnosis of nut allergy or between those with recent or distant reactions.

## Conclusions

This study has identified a complex set of strategies that nut allergic people may use to make food purchase decisions when faced with 'may contain' labelling. These may go some way to helping explain the on-going occurrence of accidental exposures to nuts which are known to occur [19]. Allergic individuals face difficult dilemmas: to avoid food that is probably safe, or to eat foods with MCL at the risk of a reaction. Of course it is not only within the shopping environment that such dilemmas exist - eating out in restaurants provides a further set of challenges [38]. We did not assess the advice that participants in this study had received from clinicians nor is it known what advice clinicians generally give, but existing data about community reactions [19] and allergen contaminants [17] suggest that in clinical settings patients should continue to be cautioned about the reality of potential reactions to food with cautionary labels. If allergic individuals decide to consume such products, they must be advised to reduce their risks by avoiding eating in 'high risk' environments e.g. in remote areas or without access to rescue medication.

It seems that from a legal perspective erring on the side of using 'may contain' labels rather than not, is the most defensible position to take [39]. However, it is vital that food producers continue to undertake risk assessment for contamination from nuts and seek to use clear 'contains' or 'does not contain' labelling wherever possible. Research is required to establish exactly what the relationship is between cross contamination with nuts and 'may contain' labelling. Finally, all those that are responsible for providing advice to nut allergic individuals need to take into account the rich range of reasoning that consumers draw on to make and justify their decisions to consume products that may contain nuts and seek to understand the inferences that they make from the presence or absence of 'may contain' labelling.

## Competing interests

The authors declare that they have no competing interests.

## Authors' contributions

All authors have participated in the design of the study and the preparation of the manuscript. JB was principal investigator with primary responsibility for the study and analysis of data and led the writing of this manuscript. JL and KM conducted all interviews and participant tasks. KG provided allergy dietetic expertise particularly for the PCRT. RS and MR provided expertise regarding labelling. MHG provided advice as an allergic consumer. JSL is an allergist and provided clinical expertise for the study design and analysis.

## Pre-publication history

The pre-publication history for this paper can be accessed here:

http://www.biomedcentral.com/1471-2458/11/734/prepub
